# Novel activity and participation scales for children, adolescents, and young adults with postacute infection and vaccination syndromes and/or ME/CFS

**DOI:** 10.1007/s00431-026-07125-9

**Published:** 2026-06-05

**Authors:** Carola Weidmann, Annika Grabbe, Maria Eberhartinger, Alissa Kircher, Ariane Leone, Cordula Warlitz, Silvia Stojanov, Uta Behrends, Lorenz L. Mihatsch

**Affiliations:** 1 Munich Chronic Fatigue Center for Young People (MCFC), Pediatrics, Technical University of Munich, TUM School of Medicine and Health, Children’s Hospital, Munich, Germany; 2 Munich Chronic Fatigue Center for Young People (MCFC), Child and Adolescent Psychosomatics, Technical University of Munich, TUM School of Medicine and Health, Children’s Hospital, Munich, Germany; 3 Division of Pediatric Psychosomatic Medicine, Department of Pediatrics and Adolescent Medicine, KJF Klinikum Josefinum, Augsburg, Germany

**Keywords:** Activity scale, Participation scale, ME/CFS, Post-COVID, Postacute infection syndromes, PAIVS

## Abstract

Children, adolescents, and young adults (CYP) with postacute infection and vaccination syndromes (PAIVS), and/or myalgic encephalomyelitis/chronic fatigue syndrome (ME/CFS), experience profound loss in activity and participation. We introduce and psychometrically validate two new brief, age-adapted, and domain-specific questionnaires for clinical use assessing activity and participation in this vulnerable patient group. For this, 91 patients (aged 10–25 years) were assessed at the Munich Chronic Fatigue Center (MCFC) from 12/2022 to 11/2024. We designed the MCFC Activity Scale and MCFC Participation Scale and assessed construct validity using confirmatory factor analysis for both questionnaires. Reliability was evaluated via Cronbach’s *α*. Factor-based MCFC Activity and Participation Scores (0–100) were derived and correlated with Bell Score, FSS, DSQ-PEM, and SF-12 Component Summary Scales (PCS and MCS). Discrimination for ME/CFS was evaluated using ROC analyses. Participants (mean age 15.6 ± 2.4 years) were predominantly female (64%). 65% were diagnosed with ME/CFS. The MCFC Activity Scale showed excellent one-factor fit (comparative fit index, CFI = 1.00) and good internal consistency (*α* = 0.82). The MCFC Participation Scale showed good internal consistency (*α* = 0.85) and acceptable one-factor fit (CFI = 0.817). Factor-based activity and participation were strongly correlated yet distinct (*r* = 0.73). Derived MCFC Activity and Participation Scores differed significantly by ME/CFS diagnosis (*p* ≤ 0.009). Scores correlated with Bell Score, FSS, DSQ-PEM, and SF-12 PCS (all *p* ≤ .002). For ME/CFS discrimination, the Activity Score achieved an AUC = 0.78 and the Participation Score an AUC = 0.72.

*Conclusion*: The Activity Scale demonstrated strong construct validity. The Participation Scale showed good internal consistency. Both scores demonstrated good convergent validity with established patient-reported outcome measures, supporting clinical utility. They may serve as pragmatic screening tools for this vulnerable patient group.

**Supplementary Information:**

The online version contains supplementary material available at 10.1007/s00431-026-07125-9.

**Table:** 

**What is Known:** • *PAIVS and ME/CFS are debilitating diseases that lead to substantial loss in daily activity and social participation.* • *Assessing limitations in activity and participation is important for clinical decision-making and disease monitoring.*	
**What is New:** • *The novel MCFC Activity and Participation Scales are brief, age-adapted, and domain-specific questionnaires assessing activity and participation in young people with PAIVS and ME/CFS.* • *Both questionnaires are valid and reliable instruments. They may serve as clinically usable tools to improve patient care in this vulnerable group.*	

## Introduction

Myalgic encephalomyelitis/chronic fatigue syndrome (ME/CFS, ICD-10 GM G93.3, and ICD-10 CM G93.32) is a chronic, complex, systemic exertion intolerance disease often triggered by acute infection affecting adults and young people alike [1–3]. It is characterized by disabling fatigue, post-exertional malaise (PEM), unrefreshing sleep, combined with cognitive dysfunction, and/or orthostatic intolerance. PEM is the cardinal clinical feature referring to a symptom worsening following daily activities that were well tolerated before disease onset and are insufficiently alleviated by rest and sleep [1–3]. With the COVID-19 pandemic, increasing numbers of individuals presented with similar symptoms following SARS-CoV-2 infection, and some were diagnosed with ME/CFS [4–7]. Here, we use the umbrella term postacute infection and vaccination syndromes (PAIVS) to summarize nonspecific chronic clinical features following infection, often dominated by PEM [8].

PAIVS and ME/CFS usually lead to a substantial loss of physical function, daily activity, and social participation and can render patients housebound or bedridden. Functional loss is one of the most consequential aspects of PAIVS and ME/CFS [9, 10]. Young people frequently withdraw from school, reduce activities, and require assistance at home [11, 12]. In severe cases, even basic activities such as personal hygiene or eating must be carefully paced to avoid PEM [13]. A structured and systematic documentation of functional loss is essential for clinical decision-making, disease monitoring, and evaluating interventions.

Currently, the Bell Score is probably the most commonly used instrument in ME/CFS to assess limitations in daily functioning [14]. While simple and familiar, each level aggregates several life domains; assumes adult role expectations; provides limited discrimination between patients with different school, work, or family responsibilities; and has never undergone psychometric evaluation.

The *Child and Adolescent Scale of Participation (CASP)* [15], *Participation and Environment Measure for Children and Youth (PEM-CY)* [16], and *Questionnaire of Young People’s Participation (QYPP)* [17] are well-established instruments to assess participation in pediatric patients. They demonstrated reliability and validity, although many studies have focused primarily on specific diagnostic groups (e.g., cerebral palsy). However, the instruments differ substantially in their normative frames: CASP rates respondents relative to same-aged peers, whereas PEM-CY and QYPP assess participation frequency and involvement without reference to normative expectations. Additionally, the instruments can be demanding to complete due to their length and are therefore inadequate for ME/CFS patients, who often have limited and fluctuating energy capacity and for whom understanding intraindividual changes from premorbid functioning levels is clinically informative.

For pediatric PAIVS and ME/CFS, five key requirements emerge: (1) age-appropriate content that reflects typical daily activities of children, adolescents, and young people (CYP); (2) separation of major life domains to allow differential assessment of impairments (e.g., participation in school, social life, and self-care); (3) inclusion of disease-related content to reflect disease severity; (4) intraindividual referencing to the premorbid level of participation, as it often falls sharply from a previously high level; and (5) brevity and low respondent burden, to facilitate efficient and feasible assessment. Currently used measurement instruments do not fully meet these requirements.

To address these needs, this study aims to introduce two brief, age-adapted questionnaires assessing activity and participation in CYP with PAIVS and/or ME/CFS, to evaluate their psychometric properties, and to develop and validate activity and participation scores for clinical use.

## Materials and methods

### Study design and population

This study included 91 CYP from the Multicenter Long COVID Registry (MLC-R) [18] and the German ME/CFS Registry (MECFS-R) [19]. Patients were referred to the Munich Chronic Fatigue Center for Young People (MCFC) at the Technical University of Munich (TUM) for evaluation of suspected PAIVS, including post-COVID condition (PCC), and/or ME/CFS between December 2022 and November 2024.

### Ethics

This registry-based study and both registries (MLC-R and MECFS-R) were approved by the Ethics Committee of the TUM School of Medicine and Health (2025–466, 2021–511, and 116–21, respectively) and adhere to the Declaration of Helsinki and its subsequent amendments. Written informed consent was obtained from patients and/or legal guardians for minors prior to inclusion in registries.

### Inclusion criteria

Inclusion criteria were written informed consent for participation in the MECFS-R and/or the MLC-R and completion of the MCFC Activity and the Participation Scales (see below).

### Collection of clinical data and patient-reported outcome measures

The following patient-reported outcome measures (PROMs) were assessed: Bell Score [14], Fatigue Severity Scale (FSS) [20], DePaul Symptom Questionnaire-PEM (DSQ-PEM) [21], Short Form-12 Health Survey–Physical (SF-12 PCS), and Mental Component Summary Scale (SF-12 MCS) [22].

### Diagnosis of PAIVS and/or ME/CFS

ME/CFS was diagnosed according to the Canadian Consensus Criteria (CCC), the Institute of Medicine (IOM) criteria, the clinical diagnostic worksheet of Rowe et al. (CDW-R), and/or the pediatric case definition of Jason et al. (PCD-J) [18, 19, 23, 24]. The definitive diagnosis was confirmed by an internal interdisciplinary, multiprofessional ME/CFS board [18, 19, 23, 24]. PCC was diagnosed according to the World Health Organization (WHO) definitions for adult and pediatric patients [25, 26]. The diagnosis of post-vaccination condition (PVC) was made analogously to the criteria for PCC.

### Development of the MCFC Activity Scale

The MCFC Activity Scale was developed on the basis of (i) the German long-term care assessment modules [27]; (ii) the International Classification of Functioning, Disability and Health: Children & Youth Version (ICF-CY) [28]; and (iii) the clinical experience of the interdisciplinary, multiprofessional MCFC team and its cooperating partners. The scale assesses activity across five key dimensions: self-care, physical activity, mental activity, social contacts, and school/training/studies/work. Each dimension is rated on a six-point Likert scale indicating the severity of impairment and is supported by concrete, age-appropriate examples.

### Development of the MCFC Participation Scale

The MCFC Participation Scale was based on (i) the activity and participation domains of the ICF-CY [28]; (ii) the German version of the CASP [29]; and (iii) the clinical expertise of the interdisciplinary, multiprofessional MCFC team. The scale comprises six dimensions: participation at home, participation outside home, access to education/work, participation in school/work, self-care, and independence in everyday life. For each dimension, respondents rate how their participation level has changed compared with their preillness level. The five-point Likert scales follow the ICF categories for grading the severity of a problem [28].

### Psychometric evaluation of MCFC Activity and MCFC Participation Scale

To examine construct validity, we conducted confirmatory factor analyses (CFA) for both questionnaires (Activity Scale: *N* = 91; Participation Scale: *N* = 89) using robust maximum likelihood estimation (Huber–White estimator). For each instrument, a one-factor model was specified. Model fit was evaluated using comparative fit index (CFI), Tucker–Lewis index (TLI), root mean square error of approximation (RMSEA), and standardized root mean residual (SRMR) (CFI/TLI ≥ 0.90, RMSEA ≤ 0.08, and SRMR ≤ 0.08 regarded as acceptable). In a subsequent structural equation model (*N* = 89), we estimated the correlation between the two latent factors. Reliability was assessed by calculating internal consistency using Cronbach’s alpha and the robust Guttman’s lambda. An Activity Score and a Participation Score were subsequently derived.

### Development of MCFC Activity and MCFC Participation Score

The score development is further described in Supplementary Material M1. Briefly, a factor score for each CFA model was computed using the Thomson regression method [30], as implemented in the *lavPredict()* function of the R package lavaan [31], and normalized to a 0–100 scale. To determine the convergent validity of both scores, Pearson correlations were calculated with PROMs. Receiver operating characteristic curve (ROC) analyses were used to analyze the discriminatory value of the Activity and Participation Scores between ME/CFS and non-ME/CFS patients.

### Other statistical analysis

Continuous variables were summarized as mean (SD) and median (IQR), and groups were compared using the Kruskal–Wallis test or a one-way Welch ANOVA, as appropriate. Categorical variables were presented as absolute and relative frequencies and compared using Fisher’s exact tests. Statistical significance was assumed for two-sided *p*-values < 0.05. Statistical analysis was performed in R, version 4.3.2 (The R Foundation for Statistical Computing, Vienna, Austria).

## Results

### Patient characteristics

This study included 91 CYP, 9/91 (10%) of whom were children (10 to 12 years), 61/91 (67%) adolescents (13 to 17 years), and 21/91 (23%) young adults (18 to 25 years). 58/91 (64%) patients were female. In 19/91 (21%), the diagnoses ME/CFS (ICD-10 GM G93.3) and PCC (ICD-10 GM U09.9!) were excluded (noME/CFS-noPCC). In 13/91 (14%) patients, ME/CFS was excluded, but PCC was diagnosed (noME/CFS-PCC), and 59/91 (65%) patients were diagnosed with confirmed or probable ME/CFS with or without PCC (ME/CFS). Patients’ characteristics are summarized in Table 1. Responses to the items of the Activity and Participation Scales were significantly different across these three patient groups (Table 2).

**Table 1:** Patient characteristics

**Characteristic**	***Overall N* = *91***^***a***^	***noME/CFS− noPCC N* = *19***^***a***^	***noME/CFS− PCC N* = *13***^***a***^	***ME/CFS N* = *59***^***a***^	***p***^*b*^
**Basic characteristics**					
Sex					0.011
Male	33/91 (36%)	10/19 (53%)	8/13 (62%)	15/59 (25%)	
Female	58/91 (64%)	9/19 (47%)	5/13 (38%)	44/59 (75%)	
Age	15.64 ± 2.41 (16.0; 14.0–17.0)	14.58 ± 2.19 (14.0; 13.0–17.0)	16.77 ± 2.62 (17.0; 15.0–19.0)	15.73 ± 2.33 (16.0; 14.0–18.0)	0.054
**Patient-reported outcome measures (PROMs)**					
Bell Score	37 ± 15 (30; 30–40)	46 ± 20 (40; 30–60)	44 ± 12 (40; 40–50)	32 ± 12 (30; 30–40)	< 0.001
FSS Score	6.35 ± 0.73 (6.56; 6.00–6.89)	6.11 ± 1.06 (6.33; 5.67–6.89)	6.09 ± 0.77 (6.22; 5.44–6.78)	6.48 ± 0.55 (6.67; 6.22–6.89)	0.123
SF-12 PCS	28 ± 11 (25; 20–33)	34 ± 13 (28; 25–47)	27 ± 9 (28; 20–34)	25 ± 9 (22; 18–31)	0.028
SF-12 MCS	46 ± 9 (48; 42–52)	46 ± 9 (49; 42–51)	46 ± 10 (46; 42–53)	45 ± 10 (48; 37–52)	0.970
DSQ-PEM: PEM screening					0.145
Negative	1/91 (1%)	0/19 (0%)	1/13 (8%)	0/59 (0%)	
Positive	90/91 (99%)	19/19 (100%)	12/13 (92%)	59/59 (100%)	
DSQ-PEM: PEM duration					< 0.001
< 1 h	3/90 (3%)	3/19 (16%)	0/13 (0%)	0/58 (0%)	
2–3 h	7/90 (8%)	4/19 (21%)	3/13 (23%)	0/58 (0%)	
4–10 h	9/90 (10%)	2/19 (11%)	4/13 (31%)	3/58 (5%)	
11–13 h	10/90 (11%)	3/19 (16%)	2/13 (15%)	5/58 (9%)	
14–23 h	11/90 (12%)	1/19 (5%)	1/13 (8%)	9/58 (16%)	
> 24 h	50/90 (56%)	6/19 (32%)	3/13 (23%)	41/58 (71%)	
**ME/CFS diagnostic criteria (MBSQ)**					
CCC fulfilled	38/87 (44%)	1/17 (6%)	0/13 (0%)	37/57 (65%)	< 0.001
IOM criteria fulfilled	55/87 (63%)	4/17 (24%)	1/13 (8%)	50/57 (88%)	< 0.001
CDW-R criteria fulfilled	31/65 (48%)	2/16 (13%)	0/8 (0%)	29/41 (71%)	< 0.001
PCD-J criteria fulfilled	26/65 (40%)	1/16 (6%)	0/8 (0%)	25/41 (61%)	< 0.001
**Diagnoses**					
ME/CFS (ICD-10 GM G93.3)					< 0.001
Excluded	32/91 (35%)	19/19 (100%)	13/13 (100%)	0/59 (0%)	
Probable	19/91 (21%)	0/19 (0%)	0/13 (0%)	19/59 (32%)	
Confirmed	40/91 (44%)	0/19 (0%)	0/13 (0%)	40/59 (68%)	
Post-COVID (ICD-10 GM U09.9!)	43/91 (47%)	0/19 (0%)	13/13 (100%)	30/59 (51%)	< 0.001
Post-VAC (ICD-10 GM U12.9!)	10/91 (11%)	3/19 (16%)	1/13 (8%)	6/59 (10%)	0.788

^*a*^*n*/*N* (%); mean ± SD (median; Q1–Q3)

^*b*^Fisher’s exact test; Kruskal-Wallis rank sum test

Abbreviations: *CCC*, Canadian Consensus Criteria; *CDW-R*, clinical diagnostic worksheet of Rowe et al.; *DSQ-PEM*, DePaul Symptom Questionnaire for Post-Exertional Malaise; *FSS*, Fatigue Severity Scale; *IOM*, Institute of Medicine; *MBSQ*, Munich Berlin Symptom Questionnaire; *PCD-J*, pediatric case definition of Jason et al.; *SF-12 MCS*, Short Form 12 Health Survey–Mental Component Summary Scale; *SF-12 PCS*, Short Form 12 Health Survey–Physical Component Summary Scale

**Table 2:** Patient responses on the MCFC Activity and MCFC Participation Scale items

**Characteristic**	***Overall N* = *91***^***a***^	***noME/CFS− noPCC N* = *19***^***a***^	***noME/CFS− PCC N* = *13***^***a***^	***ME/CFS N* = *59***^***a***^	***p***^*b*^
**Activity Scale items**					
Self-care	5.0 (5.0–6.0)	6.0 (5.0–6.0)	6.0 (6.0–6.0)	5.0 (5.0–6.0)	< 0.001
Physical activity	5.0 (4.0–5.0)	5.0 (4.0–5.0)	5.0 (5.0–5.0)	4.0 (4.0–5.0)	< 0.001
Mental activity	3.0 (2.0–5.0)	5.0 (4.0–5.0)	5.0 (4.0–5.0)	2.0 (2.0–4.0)	< 0.001
Social contacts	4.0 (4.0–5.0)	5.0 (4.0–5.0)	5.0 (5.0–6.0)	4.0 (4.0–5.0)	0.004
School/training/ studies/work	2.0 (1.0–4.0)	4.0 (2.0–5.0)	4.0 (3.0–5.0)	1.0 (1.0–4.0)	0.001
**Participation Scale items**					
Participation at home	3.0 (2.0–4.0)	4.0 (3.0–4.0)	3.0 (3.0–5.0)	3.0 (2.0–3.0)	0.018
Participation outside the home	2.0 (2.0–3.0)	2.0 (2.0–4.0)	2.0 (2.0–4.0)	2.0 (2.0–2.0)	0.025
Access to education/work	2.0 (1.0–3.0)	2.0 (1.0–3.0)	3.0 (1.0–3.0)	2.0 (1.0–2.0)	0.043
Participation in school/work	2.0 (1.0–3.0)	3.0 (1.0–4.0)	3.0 (2.0–3.0)	2.0 (1.0–3.0)	0.025
Self-care	4.0 (4.0–5.0)	5.0 (4.0–5.0)	5.0 (4.0–5.0)	4.0 (3.0–5.0)	0.014
Independence in daily life	3.0 (2.0–4.0)	4.00 (3.0–5.0)	3.50 (2.5–5.0)	3.0 (2.0–4.0)	0.005

^*a*^Median; (Q1–Q3)

^*b*^Kruskal-Wallis rank sum test

### Psychometric evaluation of the MCFC Activity and MCFC Participation Scale

**Fig. 1:**
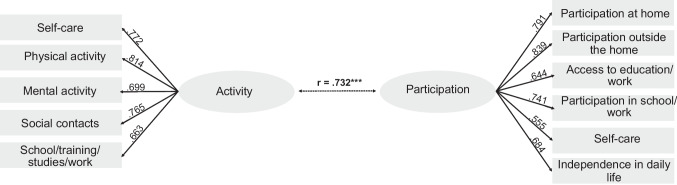
Factor loadings are depicted for both CFAs, indicating the correlation between the questionnaire items (gray rectangles) and the latent activity and latent participation scale factor (gray ellipses). The dashed double arrow between the activity and the participation factor indicates the coefficient of correlation between both factors estimated by a structural equation model.

The CFA of the Activity Scale showed a very good model fit with *χ*^2^(5) = 4.75, *p* = 0.448, CFI = 1.00, TLI = 1.003, RMSEA < 0.001 (95%-CI [0.000, 0.142]), and SRMR = 0.027. All standardized factor loadings were moderate to strong and significant (all *p* < 0.001) (Fig. 1). Additionally, Cronbach’s *α* = 0.82 and Guttman’s *λ* = 0.84 indicated good internal consistency. The CFA of the Participation Scale showed an acceptable model fit with *χ*^2^(9) = 55.83, *p* < 0.001, CFI = 0.817, TLI = 0.695, RMSEA = 0.242 (95%-CI [0.183, 0.304]), and SRMR = 0.083. The standardized factor loadings were moderate to strong and all significant (all *p* < 0.001) (Fig. 1). Again, Cronbach’s *α* = 0.85 and Guttman’s *λ* = 0.87 indicated good internal consistency. The coefficient of correlation between the two factors, estimated using a structural equation model, was *r* = 0.732 (95%-CI [0.618, 0.816]), *p* < 0.001, indicating that these are two strongly related yet distinct psychometric constructs (Fig. 1).

### MCFC Activity and MCFC Participation Score

**Fig. 2:**
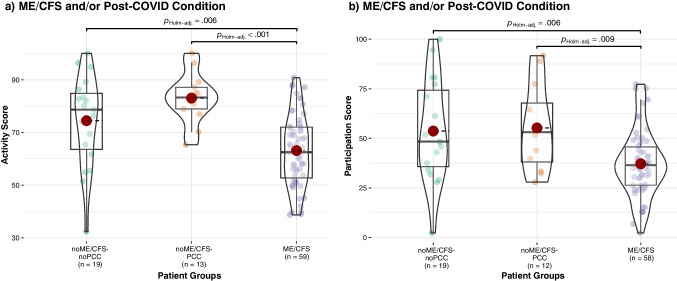
Boxplots showing the distribution of the (**a**) MCFC Activity Score and (**b**) MCFC Participation Score with individual observations are overlaid. Participants were classified as excluded ME/CFS and PCC (noME/CFS-noPCC), excluded ME/CFS with PCC (noME/CFS-PCC), and ME/CFS with or without PCC (ME/CFS). Group differences were tested using a one-way Welch ANOVA. Post- hoc pairwise comparisons were performed using the Games–Howell test for all group pairs, with Holm correction for multiple testing.

Based on the CFAs’ factor loadings, we developed the MCFC Activity and MCFC Participation Score as described in the “Materials and method” section. The scores can be calculated as shown in the Supplementary Material M1. For the MCFC Activity Score, there was no significant difference between the sexes (*p* = 0.513) nor any significant correlation with age (*r*_*Pearson*_ = 0.10, *p* = 0.342). The Activity Score was significantly different between the three patient groups: noME/CFS-noPCC, noME/CFS-PCC, and ME/CFS (*p* < 0.001, *ω*^2^ = 0.54) (Fig. 2a). In particular, ME/CFS patients showed significantly worse Activity Scores compared to both groups without ME/CFS (noME/CFS-noPCC: *p* = 0.006; post hoc and noME/CFS-PCC: *p* < 0.001; post hoc). There was no significant difference between patients with noME/CFS-noPCC and noME/CFS-PCC (*p* = 0.093; post hoc). Supplementary Figure S1 shows patient differentiation based on ME/CFS and PCC diagnoses. The results also indicate significant differences among the groups (*p* < 0.001, *ω*^2^ = 0.28). Specifically, patients with probable ME/CFS (*p* < 0.001; post hoc) or confirmed ME/CFS diagnosis (*p* < 0.001; post hoc) showed significantly worse Activity Scores than patients with excluded ME/CFS. There was no significant difference between probable ME/CFS and confirmed ME/CFS. The diagnosis of PCC (ICD-10 GM U09.9!) did not result in any differences in the Activity Score (*p* = 0.477, *g*_*Hedges*_ = − 0.15).

Analogously, there was no significant difference between the sexes for the MCFC Participation Score (*p* = 0.780), nor was there a significant correlation between the score and patients’ age (*r*_*Pearson*_ = 0.06, *p* = 0.569). Similarly, the Participation Scores significantly differed between the patient groups (*p* = 0.007, *ω*^2^ = 0.29) (Fig. 2b). Again, ME/CFS patients showed significantly worse Participation Scores than patients without ME/CFS (noME/CFS-noPCC: *p* = 0.006; post hoc and noME/CFS-PCC: *p* = 0.009; post hoc). NoME/CFS-noPCC and noME/CFS-PCC did not differ significantly. The differentiation of patients based on their diagnoses also shows significant differences in the Participation Score (*p* < 0.001, *ω*^2^ = 0.18) (Supplementary Figure S2). In patients with probable ME/CFS (*p* < 0.001; post hoc) or confirmed ME/CFS diagnoses (*p* = 0.020; post hoc), the Participation Score was significantly worse than in patients with excluded ME/CFS diagnoses, without any significant differences between patients with probable and confirmed ME/CFS diagnoses. The Participation Scores did not significantly differ between patients with and without PCC (*p* = 0.705, *g*_*Hedges*_ = 0.08).

**Fig. 3:**
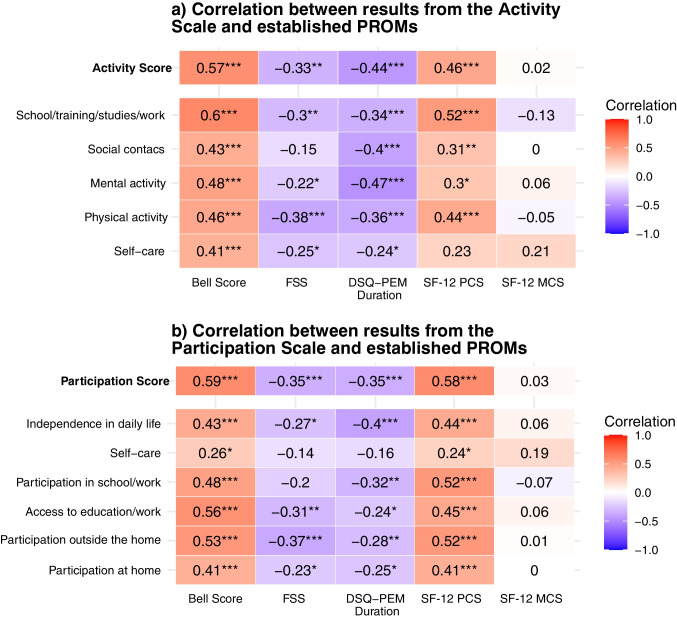
(**a**) Heatmap showing the Pearson’s coefficient of correlations between results from the MCFC Activity Score and single items of the MCFC Activity Scale with the indicated PROMs: Bell Score, Fatigue Severity Scale (FSS), DSQ-PEM (Duration), 12-item Short Form Health Survey (SF-12) physical (PCS), and mental health summary scores (MCS). (**b**) Heatmap showing the correlation between the MCFC Participation Score and single items of the MCFC Participation Scale with the indicated PROMs. Level of significance is indicated by ^*^p < 0.05, ^**^p < 0.01, and ^***^p < 0.001.

Both scores significantly correlated with clinically relevant PROMs, showing convergent validity (Fig. 3). Specifically, both scores showed moderate to high correlation with Bell Score (*p* < 0.001), FSS (*p* ≤ 0.002), DSQ-PEM (*p* < 0.001), and SF-12 PCS (*p* < 0.001). For the Activity Scale, most items correlated moderately to strongly with most PROMs. The largest correlation was between the item school/training/studies/work and Bell Score (*r* = 0.60, *p* < 0.001). For the Participation Scale, most items correlated weakly to moderately with most PROMs. The largest correlation was between access to education/work and Bell Score (*r* = 0.56, *p* < 0.001). There was no significant correlation between any of the activity or participation items and the SF-12 MCS.

To assess the discriminatory value of the Activity and the Participation Score for the diagnosis ME/CFS, ROC analyses were employed. Table 3 shows the optimal cut-off values for both scores in comparison to the Bell Score. The Activity Score discriminated better than the Bell Score in area under the curve (AUC), accuracy, and sensitivity. The Participation Score showed slightly lower discriminatory performance than the Bell Score, although the differences were small.

**Table 3:** ROC analysis and optimal cut-off values

	**Activity Score**	**Participation Score**	**Bell Score**
AUC	0.78	0.72	0.75
Optimal threshold^a^	73.689	42.956	35
Accuracy	0.77	0.70	0.73
Sensitivity	0.80	0.71	0.71
Specificity	0.72	0.68	0.75

^a^Optimal threshold was determined by the Youden-index

Abbreviations: *AUC*, area under the curve

## Discussion

This study introduced the clinically actionable MCFC Activity and MCFC Participation Scales. In a psychometric validation in pediatric patients with PAIVS and/or ME/CFS, the Activity and Participation Scales showed supportive evidence of reliability and validity. Accordingly, scores derived from those questionnaire items provided solid and clinically usable tools to improve patient care.

### Development of the questionnaires

We developed the MCFC Activity Scale, which assesses activity across five dimensions. This scale was designed to be concise, age-adapted, and domain-specific, thereby addressing limitations of global functional measures such as the Bell Score, which do not differentiate between major life domains. Additionally, we developed the MCFC Participation Scale, which evaluates disease-related restrictions in participation across six life domains. This scale was designed to capture clinically relevant participation restrictions in ME/CFS, including limitations in school attendance, the need for care, and loss of independence [32–35]. Both questionnaires were designed to be completed within a few minutes, easily understood, and suitable for screening and serial monitoring. They have been integrated into clinical practice at our institution and into the core set of multicenter registries [18, 19]. This reflects their feasibility and practical uptake, while their psychometric properties are evaluated separately below.

### Psychometric evaluation of the questionnaires

The mean values for activity and participation varied across different life domains. Notably, self-care remained largely possible, whereas school attendance and work were severely restricted. This finding is consistent with previous research showing that ME/CFS is often associated with school absence [33, 36], highlighting the importance of support measures in this context. Additionally, this finding underscores a methodological limitation of the Bell Score: it does not differentiate across various life domains.

Regarding construct validation, factors of both CFA models, for the Activity and Participation Scale, loaded moderately to strongly onto a single latent factor, confirming the hypothesized underlying construct. Together with the model fit indices that showed a good fit to data structure for the Activity Scale and an acceptable model fit for the Participation Scale, the overall construct validity was good for the Activity Scale and moderate for the Participation Scale. The high reliability values (activity: *α* = 0.82, *λ* = 0.84; participation: *α* = 0.85, *λ* = 0.87) demonstrated that both models exhibit high internal consistency. Overall, this confirms that both scales are valid and reliable instruments.

Although not surprising, the factors derived from the Activity and Participation Scales were well correlated (*r* = 0.73), but not sufficiently high to make the questionnaires interchangeable. In fact, activity and participation address two different aspects of clinical care. The Activity Scale is particularly relevant for medical and rehabilitative purposes, such as selecting an appropriate diagnostic and therapeutic setting, preventing PEM, and evaluating disease progression. The Participation Scale focuses on social care and medical assistance, including the selection and evaluation of appropriate support measures.

### Psychometric evaluation of the scores

The Activity and Participation Scores allow examination and interpretation of results through a single metric. Using the Thomson regression method, factor loadings, error variances, and factor variances were incorporated into item weighting [30]. Therefore, the scores are more differentiated than a simple sum of the items. The scores validly differentiated between patients with and without ME/CFS. There was also no difference in scores regarding the presence of a PCC diagnosis. This supports evidence for the known-groups validity of both instruments in this cohort. It also suggests that the instruments capture aspects of disease burden that are closely related to ME/CFS-associated functional impairment. In addition, both scores correlated significantly with established PROMs commonly used to assess functional status and disease severity, further supporting their convergent validity. However, these findings should be interpreted as evidence that the instruments reflect severity-related impairment, rather than as validation of distinct severity strata.

The factors resulting from both scales correlated better with each other (*r* = 0.73) than with results from other instruments and thus measured closely related but not redundant features. In contrast, the Bell Score correlates with both Activity (*r* = 0.57) and Participation Score (*r* = 0.59) at similar levels, suggesting that it measures both domains without distinguishing between them. Further examination of the subscales reveals that fatigue severity (measured by FSS) was particularly associated with limitations in activities outside the home, whereas results from DSQ-PEM were primarily associated with levels of cognitive function and social contacts. The SF-12 PCS results were related to most domains but showed minimal correlation with self-care, suggesting that it provides little information about self-care capacity. In contrast, the SF-12 MCS results showed no correlations with other life domains but were, although not significantly, most closely associated with self-care. The ability to perform personal care appears to have a particularly important impact on mental status.

In both scores, the results for the school/work subdomains showed particularly strong associations with other PROMs, highlighting their relevance as indicators of disease burden and functional impairment. This underscores the high relevance of education and work issues for these patient groups [2, 37, 38] and indicates an important special need in psychosocial care. Our findings indicate that assessing activity and participation across various domains is helpful to adequately address all clinically relevant aspects and facilitates holistic approaches to diagnostics and treatment.

Besides the importance of examining various life domains, it seems useful to summarize them into a single score. The interpretation of results might be supported by using a threshold. For example, with further validation, this threshold might serve as a future clinical screening tool, enabling the stratification of patients to ensure appropriate care. In this exploratory analysis, the Activity Score outperforms the Bell Score in AUC, accuracy, and sensitivity, thereby allowing better discrimination between ME/CFS and no ME/CFS, further supporting their validity. However, limitations in activity and participation are not specific to PAIVS and/or ME/CFS and may occur in other chronic conditions as well. Thus, the levels of sensitivity and specificity of both our instruments might depend on the type and severity of diseases included in the non-ME/CFS group. Overall, the MCFC Activity Score provides a more comprehensive score compared to the Bell Score and offers a better insight into limitations within distinct life domains.

### Strength

Both instruments are brief, domain-specific, and have a low assessment burden, which makes them clinically feasible for assessing activity and participation in CYP with PAIVS and/or ME/CFS, including severely affected patients with limited energy capacity. Their domain-specific structure allows activity and participation restrictions to be described separately across clinically relevant life domains, while the derived overall scores provide concise summary measures for clinical documentation and research. The psychometric evaluation was performed in a well-characterized pediatric patient cohort assessed in a specialized PAIVS and ME/CFS clinic, including patients with and without ME/CFS.

### Limitations

Several limitations must be acknowledged. First, the single-center data may have introduced selection and spectrum bias. Therefore, results may not be generalizable to other cohorts. Due to their disease-specificity by design, a generalization to other (acute/postacute onset) chronic conditions remains to be evaluated. Second, no healthy control group was included. Consequently, normative values could not be established, and score interpretation is currently limited to comparisons within this clinical cohort. Third, the non-ME/CFS comparison groups were relatively small, limiting the power of their analyses, including the group comparisons, the precision of ROC analyses, and the proposed thresholds. The suggested cutoff values should therefore be regarded as exploratory and warranting external validation. Fourth, the Participation Scale showed weaker CFA model fit than the Activity Scale, indicating more limited evidence for construct validity. Although internal consistency and correlations with PROMs supported its clinical relevance, further validation against established participation instruments is needed. Finally, although both scales differentiated between diagnostic groups and correlated with established PROMs reflecting disease burden and impairment, this study did not validate a formal disease severity stratification. Further studies should evaluate generalizability, normative values, longitudinal responsiveness, and potential use for severity classification.

## Conclusion

We have introduced two novel scales for assessing activity and participation in pediatric PAIVS and ME/CFS patients. Overall, we provide supportive psychometric evidence for both instruments, with strong evidence for the MCFC Activity Scale and moderate evidence for the MCFC Participation Scale. The derived scores may provide pragmatic, clinically usable tools for structured assessment and monitoring, warranting further validation.

## Supplementary Information

Below is the link to the electronic supplementary material.

ESM 1 (PDF.606 KB)

ESM 2 (PDF.66.6 KB)

ESM 3 (PDF.333 KB)

ESM 4 (PDF.340 KB)

## Data Availability

All data supporting the results are included in the manuscript as well as its supplementary material.

## Abbreviations

AUC: Area under the curve
CASP: Child and Adolescent Scale of Participation
CCC: Canadian Consensus Criteria
CDW-R: Clinical diagnostic worksheet of Rowe et al.
CFA: Confirmatory factor analysis
CFI: Comparative fit index
CYP: Children and young people
DSQ-PEM: DePaul Symptom Questionnaire for Post-Exertional Malaise
FSS: Fatigue Severity Scale
ICD-10 CM: International Classification of Diseases, 10th Revision, Clinical Modification
ICD-10 GM: International Classification of Diseases, 10th Revision, German Modification
ICF-CY: International Classification of Functioning, Disability and Health: Children & Youth Version
IOM: Institute of Medicine
MCFC: Munich Chronic Fatigue Center for Young People
ME/CFS: Myalgic encephalomyelitis/chronic fatigue syndrome
MECFS-R: German Registry for ME/CFS
MLC-R: Multicenter Long COVID Registry
NPV: Negative predictive value
PAIVS: Postacute infection and vaccination syndrome
PCC: Post-COVID condition
PCD-J: Pediatric case definition of Jason et al.
PEM: Post-exertional malaise
PEM-CY: Participation and Environment Measure for Children and Youth
PPV: Positive predictive value
PROM: Patient-reported outcome measure
PVC: Post-vaccination condition
QYPP: Questionnaire of Young People’s Participation
RMSEA: Root mean square error of approximation
ROC: Receiver operating characteristic
SF-12 MCS: Short Form-12 Health Survey–Mental Component Summary Scale
SF-12 PCS: Short Form-12 Health Survey–Physical Component Summary Scale
SRMR: Standardized root mean residual
TLI: Tucker–Lewis index
TUM: Technical University of Munich
WHO: World Health Organization
