# Supplementary material for: Novel activity and participation scales for children, adolescents, and young adults with postacute infection and vaccination syndromes and/or ME/CFS
Source: Eur J Pediatr. 2026 Jun 5;185(7):471. doi: 10.1007/s00431-026-07125-9 (PMC13241460; doi:10.1007/s00431-026-07125-9)
Supplement: Supplementary file 1 — (PDF.606 KB) [file 431_2026_7125_MOESM1_ESM.pdf]

Supplementary Figures – European Journal of Pediatrics

Novel Activity and Participation Scales for Children, Adolescents, and Young Adults with Post-Acute Infection and Vaccination Syndromes and/or ME/CFS

Carola Weidmann[1], Annika Grabbe[1], Maria Eberhartinger[1], Alissa Kircher[1], Ariane Leone[1], Cordula Warlitz[1], Silvia Stojanov[2,3], Uta Behrends[1], Lorenz L. Mihatsch[1]

[1] Technical University of Munich, Germany; TUM School of Medicine and Health, Munich Chronic Fatigue Center for Young People (MCFC), Pediatrics, Children's Hospital, Munich, Germany.
[2] Technical University of Munich, Germany; TUM School of Medicine and Health, Munich Chronic Fatigue Center for Young People (MCFC), Child and Adolescent Psychosomatics, Children's Hospital, Munich, Germany.
[3] Division of Pediatric Psychosomatic Medicine, Department of Pediatrics and Adolescent Medicine, KJF Klinikum Josefinum, Augsburg, Germany.

Corresponding Author:
Lorenz L. Mihatsch
TUM University Hospital, Department of Pediatrics
Technical University of Munich
TUM School of Medicine and Health
Parzivalstraße 16
80804 Munich, Germany
Tel. +48 89 3068 2439
l.mihatsch@tum.de

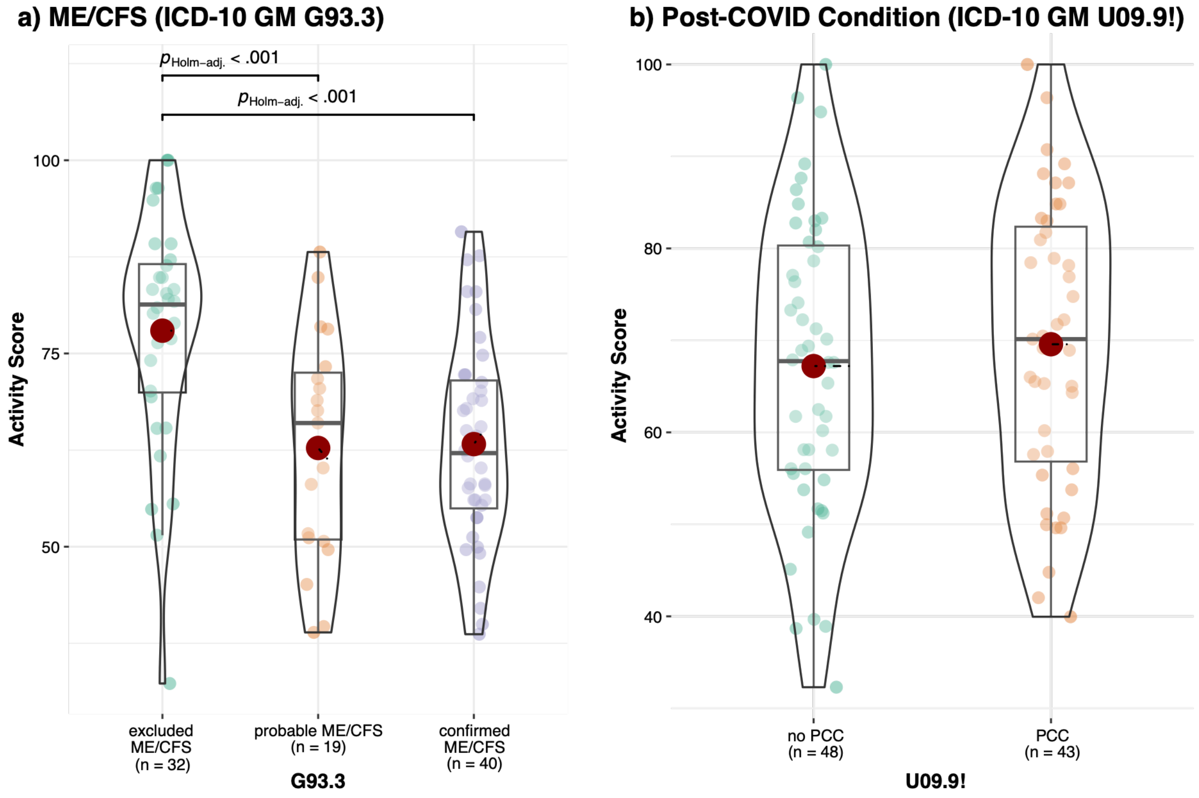

Suppl. Fig. S1 ***Distribution of the MCFC Activity Scores across diagnosis groups***
Participants were classified as (a) excluded ME/CFS, probable ME/CFS, and confirmed ME/CFS, or as (b) with (PCC) or without (no PCC) post-COVID condition. Individual observations are overlaid. Group differences were tested using a one-way Welch ANOVA. Post-hoc pairwise comparisons were performed using the Games-Howell test for all group pairs, with Holm correction for multiple testing.

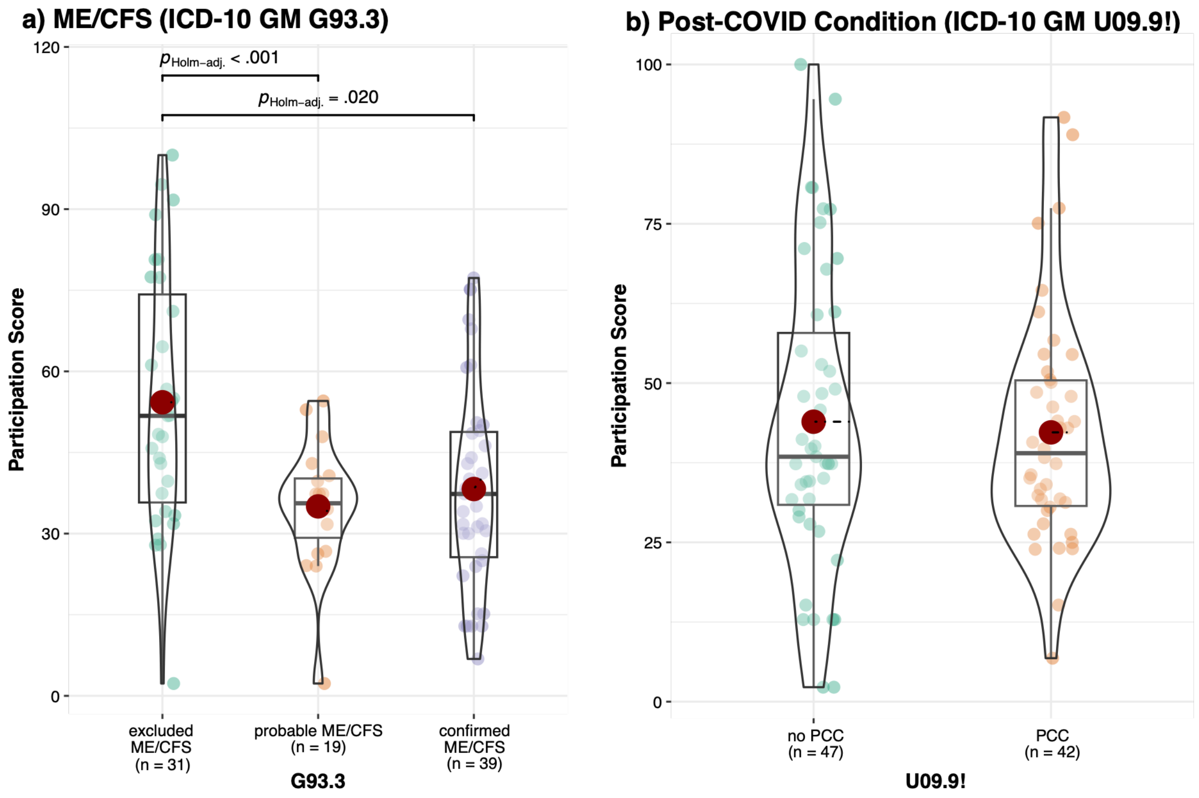

Suppl. Fig. S2 ***Distribution of the MCFC Participation Scores across diagnosis groups***
Participants were classified as (a) excluded ME/CFS, probable ME/CFS, and confirmed ME/CFS, or as (b) with (PCC) or without (no PCC) post-COVID condition. Individual observations are overlaid. Group differences were tested using a one-way Welch ANOVA. Post-hoc pairwise comparisons were performed using the Games-Howell test for all group pairs, with Holm correction for multiple testing.
