# Supplementary material for: Novel activity and participation scales for children, adolescents, and young adults with postacute infection and vaccination syndromes and/or ME/CFS
Source: Eur J Pediatr. 2026 Jun 5;185(7):471. doi: 10.1007/s00431-026-07125-9 (PMC13241460; doi:10.1007/s00431-026-07125-9)
Supplement: Supplementary file 2 — (PDF.66.6 KB) [file 431_2026_7125_MOESM2_ESM.pdf]

Supplementary Material M1 – European Journal of Pediatrics

Novel Activity and Participation Scales for Children, Adolescents, and Young Adults with Post-Acute Infection and Vaccination Syndromes and/or ME/CFS

Carola Weidmann[1], Annika Grabbe[1], Maria Eberhartinger[1], Alissa Kircher[1], Ariane Leone[1], Cordula Warlitz[1], Silvia Stojanov[2,3], Uta Behrends[1], Lorenz L. Mihatsch[1]

[1] Technical University of Munich, Germany; TUM School of Medicine and Health, Munich Chronic Fatigue Center for Young People (MCFC), Pediatrics, Children's Hospital, Munich, Germany.
[2] Technical University of Munich, Germany; TUM School of Medicine and Health, Munich Chronic Fatigue Center for Young People (MCFC), Child and Adolescent Psychosomatics, Children's Hospital, Munich, Germany.
[3] Division of Pediatric Psychosomatic Medicine, Department of Pediatrics and Adolescent Medicine, KJF Klinikum Josefinum, Augsburg, Germany.

Corresponding Author:
Lorenz L. Mihatsch
TUM University Hospital, Department of Pediatrics
Technical University of Munich
TUM School of Medicine and Health
Parzivalstraße 16
80804 Munich, Germany
Tel. +48 89 3068 2439
l.mihatsch@tum.de

A) Development of MCFC Activity and MCFC Participation Score

First, factor scores for each of the two CFA models were computed using the Thomson regression method. This method accounts for both factor loadings and measurement error, yielding an unbiased estimate of the latent variable. For each person i, the score f_i is obtained as a weighted linear combination of the centered item values $(x_i - \mu)$, with the weights reflecting the item's factor loading Λ, its error variance Θ, and the factor variance Φ, i.e.

$$f_i = W(x_i - \mu) \text{ with } W = \Phi\Lambda'(\Lambda\Phi\Lambda' + \Theta)^{-1}.$$

To enable interpretability, the scores f_i were min-max normalized, yielding a scale ranging from 0 to 100. For this, the minimum f_{min} and maximum scores f_{max} were determined for each scale by systematically combining all possible combinations of item responses. The normalized scores of the i-th individual are thus calculated by

$$normalized\ score_i = \frac{(f_i - f_{min})}{(f_{max} - f_{min})} \times 100$$

where $f_{min} = -4.046622$ and $f_{max} = 1.875503$ for the Activity Score, and $f_{min} = -1.939712$ and $f_{max} = 2.555794$ for the Participation Score.

B) Full Model Report for MCFC Activity Scale CFA-Model

Standardized Factor Loadings:

	est.std
Self-care	0.772
Physical activity	0.814
Mental activity	0.699
Social contacts	0.765
School/training/studies/work	0.663

Latent Variables:

	Estimate	Std.Err	z-value	P(>|z|)	Std.lv	Std.all
Activity =~						
Self-care	0.680	0.086	7.946	0.000	0.680	0.772
Physical activity	0.608	0.061	9.973	0.000	0.608	0.814
Mental activity	1.027	0.119	8.604	0.000	1.027	0.699
Social contacts	0.720	0.077	9.355	0.000	0.720	0.765
School/training/studies/work	1.126	0.150	7.489	0.000	1.126	0.663

Variances:

	Estimate	Std.Err	z-value	P(>|z|)	Std.lv	Std.all
Self-care	0.313	0.064	4.906	0.000	0.313	0.403
Physical activity	0.188	0.047	4.023	0.000	0.188	0.337
Mental activity	1.027	0.199	5.546	0.000	1.027	0.699
Social contacts	0.720	0.066	5.541	0.000	0.720	0.765
School/training/studies/work	1.616	0.257	6.278	0.000	1.616	0.560
Activity	1.000				1.000	1.000

C) Full Model Report for MCFC Participation Scale CFA-Model

Standardized Factor Loadings:

	est.std
Participation at home	0.791
Participation outside the home	0.839
Access to education/work	0.644
Participation in school/work	0.741
Self-care	0.555
Independence in daily life	0.684

Latent Variables:

	Estimate	Std.Err	z-value	P(>|z|)	Std.lv	Std.all
Participation =~						
Participation at home	0.809	0.095	8.541	0.000	0.809	0.791
Participation outside the home	0.766	0.102	7.476	0.000	0.766	0.839
Access to education/work	0.699	0.153	4.563	0.000	0.699	0.644
Participation in school/work	0.974	0.150	6.483	0.000	0.974	0.741
Self-care	0.525	0.083	6.355	0.000	0.525	0.555
Independence in daily life	0.841	0.098	8.557	0.000	0.841	0.684

Variances:

	Estimate	Std.Err	z-value	P(>|z|)	Std.lv	Std.all
Participation at home	0.392	0.095	4.138	0.000	0.392	0.375
Participation outside the home	0.247	0.077	3.231	0.000	0.247	0.296
Access to education/work	0.688	0.166	4.143	0.000	0.688	0.585
Participation in school/work	0.777	0.195	3.984	0.000	0.777	0.450
Self-care	0.619	0.105	5.900	0.000	0.619	0.692
Independence in daily life	0.803	0.143	5.615	0.000	0.803	0.532
Participation	1.000				1.000	1.000

D) Calculation of MCFC Activity and MCFC Participation Scores

Parameters for the Calculation

Activity Scale:

	item mean μ_j	weights w_j
Self-care	μ_1 = 5.165	w_1 = 0.286165
Physical activity	μ_2 = 4.527	w_2 = 0.425722
Mental activity	μ_3 = 3.341	w_3 = 0.122514
Social contacts	μ_4 = 4.538	w_4 = 0.258364
School/training/studies/work	μ_5 = 2.659	w_5 = 0.091660

Participation Scale:

	item mean μ_j	weights w_j
Participation at home	μ_1 = 3.146	w_1 = 0.248668
Participation outside the home	μ_2 = 2.348	w_2 = 0.373398
Access to education/work	μ_3 = 2.056	w_3 = 0.122333
Participation in school/work	μ_4 = 2.270	w_4 = 0.151111
Self-care	μ_5 = 4.124	w = 0.102240
Independence in daily life	μ_6 = 3.079	w_6 = 0.126128

Let x_{ij} be the answer to the j-th item on the Activity or Participation Scale of the i-th individual. The Activity or Participation Score f_i is than calculated by:

$$f_i = \sum_{j=1}^{n} w_j(x_{ij} - \mu_j)$$

where μ are the items' means, w the items' weights, and n the number of items in the Activity (n = 5) and Participation Scale ($n = 6$), respectively. The min-max normalization, yielding a scale ranging from 0 to 100, is calculated by

$$normalized\ score_i = \frac{(f_i - f_{min})}{(f_{max} - f_{min})} \times 100$$

where $f_{min} = -4.046622$ and $f_{max} = 1.875503$ for the Activity Score, and $f_{min} = -1.939712$ and $f_{max} = 2.555794$ for the Participation Score.

For example, if $x_i = (6, 5, 5, 6, 5)$ the f_i for the Activity Score is calculated as

$$f_i = \sum_{j=1}^{5} w_j(x_{ij} - \mu_j)$$

$$= 0.286165(6 - 5.165) + 0.425722(5 - 4.527) + 0.122514(5 - 3.341) + 0.258364(6 - 4.538) + 0.091660(5 - 2.659)$$

$$= 1.23586924,$$

whereas the

$$normalized\ score_i = \frac{(f_i - f_{min})}{(f_{max} - f_{min})} \times 100$$

$$= \frac{(1.23586924 - (-4.046622))}{(1.875503 - (-4.046622))} \times 100$$

$$= 89.1992526.$$

Thus, the Activity Score is 89.2. The Participation Score can be calculated analogously using the above values.
