# Supplementary material for: Novel activity and participation scales for children, adolescents, and young adults with postacute infection and vaccination syndromes and/or ME/CFS
Source: Eur J Pediatr. 2026 Jun 5;185(7):471. doi: 10.1007/s00431-026-07125-9 (PMC13241460; doi:10.1007/s00431-026-07125-9)
Supplement: Supplementary file 3 — (PDF.333 KB) [file 431_2026_7125_MOESM3_ESM.pdf]

Supplementary Material M2 – European Journal of Pediatrics

Novel Activity and Participation Scales for Children, Adolescents, and Young Adults with Post-Acute Infection and Vaccination Syndromes and/or ME/CFS

Carola Weidmann[1], Annika Grabbe[1], Maria Eberhartinger[1], Alissa Kircher[1], Ariane Leone[1], Cordula Warlitz[1], Silvia Stojanov[2,3], Uta Behrends[1], Lorenz L. Mihatsch[1]

[1] Technical University of Munich, Germany; TUM School of Medicine and Health, Munich Chronic Fatigue Center for Young People (MCFC), Pediatrics, Children's Hospital, Munich, Germany.

[2] Technical University of Munich, Germany; TUM School of Medicine and Health, Munich Chronic Fatigue Center for Young People (MCFC), Child and Adolescent Psychosomatics, Children's Hospital, Munich, Germany.

[3] Division of Pediatric Psychosomatic Medicine, Department of Pediatrics and Adolescent Medicine, KJF Klinikum Josefinum, Augsburg, Germany.

Corresponding Author:

Lorenz L. Mihatsch

TUM University Hospital, Department of Pediatrics

Technical University of Munich

TUM School of Medicine and Health

Parzivalstraße 16

80804 Munich, Germany

Tel. +48 89 3068 2439

l.mihatsch@tum.de

MCFC
Münchner Chronische Fatigue
Centrum für junge Menschen

TUM Universitäts Klinikum

MCFC Activity Scale

For assessing the Activity of Patients with PAIVS and/or ME/CFS

Patient:
Date of assessment:

Please tick **only one box** in each column.

Select the box that best describes what is possible in your **current** condition.

Score	Self-Care	Physical Activity	Mental Activity	Social Contacts	School/Training/Studies/Work
6	☐ I can clean/tidy my room independently.	☐ I can do sports/exercise.	☐ I can study and solve problems without difficulty.	☐ I can take part in group activities.	☐ Full day attendance is possible.
5	☐ I can shower on my own.	☐ I can leave the house and e.g. go for a walk or run errands.	☐ I can study for a few hours.	☐ I can visit someone.	☐ A maximum of 6 hours per day is possible.
4	☐ I can dress myself.	☐ I can move around the house (without assistive devices).	☐ I can read for one hour.	☐ I can receive visitors.	☐ A maximum of 4 hours per day is possible.
3	☐ I can go to the toilet on my own.	☐ I can only move with assistive devices (e.g., walker/wheelchair).	☐ I can read and remember the content.	☐ I can communicate online.	☐ A maximum of 2 hours per day is possible.
2	☐ I can eat on my own.	☐ I can only lie in bed and change position.	☐ I can read and understand the content.	☐ I can say a few words.	☐ A maximum of 1 hours per day is possible.
1	☐ Food/fluid intake is only possible with assistance.	☐ I can only lie in bed.	☐ I can read for less than 5 minutes.	☐ I can understand simple spoken messages.	☐ No attendance is possible.

MCFC Activity Scale, created by A. Grabbe, A. Leone, A. Kircher, M. Eberhartinger, C. Weidmann, S. Stojanov, U. Behrends ©

MCFC
Münchner Chronische Fatigue
Centrum für junge Menschen

TUM | Universitäts Klinikum

MCFC Participation Scale

For assessing the Participation of Patients with PAIVS and/or ME/CFS

Patient:

Date of assessment:

Assessment with patient alone ☐
Assessment with patient and accompanying person(s) ☐
Assessment with accompanying person(s) only ☐

Please evaluate how your participation has changed compared to before the illness.

Use the following response categories:

- **Unchanged:** Participation is still equally possible (5)
- **Slightly limited:** Participation is slightly less possible (4)
- **Moderately limited:** Participation is partly less possible (3)
- **Severely limited:** Participation is much less possible (2)
- **Unable**: Participation is no longer possible (1)

	Unchanged	Slightly limited	Moderately limited	Severely limited	Unable
Participation at home<br>Leisure activities (family, friends), communication and mobility at home	☐	☐	☐	☐	☐
Participation outside the home<br>Leisure activities (family, friends), communication and mobility outside the home	☐	☐	☐	☐	☐
Access to education / work<br>Participation in classes (in-person, online, homework, home-schooling), ability to work	☐	☐	☐	☐	☐
Participation in school / work<br>Interaction with classmates/colleagues, interaction during class/workday, mobility to and within school/work	☐	☐	☐	☐	☐
Self-care<br>Eating, personal hygiene, dressing	☐	☐	☐	☐	☐
Independence in daily life<br>Planning and adapting daily routine, scheduling and attending appointments, household activities, shopping, using public transport	☐	☐	☐	☐	☐

MCFC Participation Scale, created by M. Eberhartinger, C. Weidmann ©
