# Supplementary material for: Novel activity and participation scales for children, adolescents, and young adults with postacute infection and vaccination syndromes and/or ME/CFS
Source: Eur J Pediatr. 2026 Jun 5;185(7):471. doi: 10.1007/s00431-026-07125-9 (PMC13241460; doi:10.1007/s00431-026-07125-9)
Supplement: Supplementary file 4 — (PDF.340 KB) [file 431_2026_7125_MOESM4_ESM.pdf]

Supplementary Material M3 – European Journal of Pediatrics

Novel Activity and Participation Scales for Children, Adolescents, and Young Adults with Post-Acute Infection and Vaccination Syndromes and/or ME/CFS

Carola Weidmann[1], Annika Grabbe[1], Maria Eberhartinger[1], Alissa Kircher[1], Ariane Leone[1], Cordula Warlitz[1], Silvia Stojanov[2,3], Uta Behrends[1], Lorenz L. Mihatsch[1]

[1] Technical University of Munich, Germany; TUM School of Medicine and Health, Munich Chronic Fatigue Center for Young People (MCFC), Pediatrics, Children's Hospital, Munich, Germany.
[2] Technical University of Munich, Germany; TUM School of Medicine and Health, Munich Chronic Fatigue Center for Young People (MCFC), Child and Adolescent Psychosomatics, Children's Hospital, Munich, Germany.
[3] Division of Pediatric Psychosomatic Medicine, Department of Pediatrics and Adolescent Medicine, KJF Klinikum Josefinum, Augsburg, Germany.

Corresponding Author:
Lorenz L. Mihatsch
TUM University Hospital, Department of Pediatrics
Technical University of Munich
TUM School of Medicine and Health
Parzivalstraße 16
80804 Munich, Germany
Tel. +48 89 3068 2439
l.mihatsch@tum.de

MCFC
Münchner Chronische Fatigue
Centrum für junge Menschen

TUM | Universitäts Klinikum

MCFC Aktivitätsskala

Zur Erhebung der Aktivität von PatientInnen mit PAIVS und/oder ME/CFS

PatientIn:
Datum der Erhebung:

Bitte kreuze in jeder Spalte **nur ein Kästchen** an.

Wähle das Kästchen, das beschreibt, was in Deiner **aktuellen** Verfassung maximal möglich ist.

Punkte	Selbstversorgung	Körperliche Aktivität	Geistige Aktivität	Soziale Kontakte	Schule / Ausbildung / Studium / Beruf
6	☐ Ich kann mein Zimmer selbstständig saubermachen / aufräumen.	☐ Ich kann Sport treiben.	☐ Ich kann problemlos lernen und Aufgaben lösen.	☐ Ich kann in einer Gruppe etwas unternehmen.	☐ Ganztags möglich
5	☐ Ich kann mich alleine duschen.	☐ Ich kann das Haus verlassen und z.B. Spazieren gehen / etwas erledigen.	☐ Ich kann wenige Stunden lernen.	☐ Ich kann jemanden besuchen.	☐ Maximal 6 Std/Tag möglich
4	☐ Ich kann mich alleine anziehen.	☐ Ich kann mich im Haus (ohne Hilfsmittel) umherbewegen.	☐ Ich kann eine Stunde lesen.	☐ Ich kann Besucher empfangen.	☐ Maximal 4 Std/Tag möglich
3	☐ Ich kann alleine auf die Toilette gehen.	☐ Ich kann mich nur mit Hilfsmitteln (z.B. Rollator / Rollstuhl) fortbewegen.	☐ Ich kann lesen und den Inhalt erinnern.	☐ Ich kann online kommunizieren.	☐ Maximal 2 Std/Tag möglich
2	☐ Ich kann alleine essen.	☐ Ich kann nur im Bett liegen und die Position wechseln.	☐ Ich kann lesen und den Inhalt verstehen.	☐ Ich kann ein paar Worte sprechen.	☐ Maximal 1 Std/Tag möglich
1	☐ Nahrungs-/ Flüssigkeits-aufnahme nur mit Hilfe möglich.	☐ Ich kann nur im Bett liegen.	☐ Ich kann weniger als 5 Minuten lesen.	☐ Ich kann einfache gesprochene Mitteilungen verstehen.	☐ Keine Teilnahme möglich

MCFC Aktivitätsskala, erstellt von A. Grabbe, A. Leone, A. Kircher, M. Eberhartinger, C. Weidmann, S. Stojanov, U. Behrends ©

Münchner Chronische Fatigue
Centrum für junge Menschen

Universitäts Klinikum

MCFC Teilhabeskala

Zur Erhebung der Teilhabe von PatientInnen mit PAIVS und/oder ME/CFS

PatientIn:

Datum der Erhebung:

Erhebung mit PatientIn alleine ☐
Erhebung mit PatientIn und Begleitperson(en) ☐
Erhebung mit Begleitperson(en) ☐

Bitte schätze ein, wie sich deine Teilhabe im Vergleich zu vor der Erkrankung verändert hat.

Benutze dazu die folgenden Antwortkategorien:

- **Unverändert:** Eine Beteiligung ist noch gleichermaßen möglich (5)
- **Etwas eingeschränkt:** Eine Beteiligung ist etwas weniger möglich (4)
- **Mittelmäßig eingeschränkt:** Eine Beteiligung ist teilweise weniger möglich (3)
- **Sehr eingeschränkt:** Eine Beteiligung ist viel weniger möglich (2)
- **Nicht in der Lage**: Eine Beteiligung ist nicht mehr möglich (1)

	Unverändert	Etwas eingeschränkt	Mittelmäßig eingeschränkt	Sehr eingeschränkt	Nicht mehr in der Lage
Beteiligung zu Hause<br>Freizeitaktivitäten (Familie, Freunde), Kommunikation und Fortbewegung zu Hause	☐	☐	☐	☐	☐
Beteiligung außer Haus<br>Freizeitaktivitäten (Familie, Freunde), Kommunikation und Fortbewegung außer Haus	☐	☐	☐	☐	☐
Zugang zu Bildung / Arbeit<br>Teilnahme am Unterricht (Präsenz, online, Hausaufgaben, -unterricht), Arbeitsfähigkeit	☐	☐	☐	☐	☐
Beteiligung in Schule / Beruf<br>Interaktion mit Mitschüler/ Kollegen, Interaktion im Unterricht/ Arbeitsalltag, Fortbewegung in und zur Schule/ Arbeit	☐	☐	☐	☐	☐
Selbstversorgung<br>Nahrungsaufnahme, Körperpflege, Ankleiden	☐	☐	☐	☐	☐
Selbstständigkeit im Alltag<br>Tagesablauf planen und anpassen, Termine planen und durchführen, Haushaltsaktivitäten, Einkäufe, Öffentliche Verkehrsmittel nutzen	☐	☐	☐	☐	☐

MCFC Teilhabeskala, erstellt von M. Eberhartinger, C. Weidmann ©
